# Therapeutic Notes

**Published:** 1896-04-04

**Authors:** 


### Therapeutic Notes.

### THE THERAPEUTIC VALUE OF IODATES.

The physiological value of iodate of quinine was insisted upon many years ago by Sir Charles Cameron, and from time to time the clinical use of other iodates has been recommended by other authorities. It has, however, been left for Dr. Ruhemann,* of Berlin, to deal collectively with the therapeutic value of the different iodates which are now, thanks to improved chemical methods, reliable and trustworthy chemical bodies. The iodates with which he deals in his excellent paper include those of silver, strontium, zinc, mercury, lithium, quinine, strychnine, codeine, hyoscine, atropine. The first in this list is recommended as an excellent internal astringent and antiseptic, valuable in cases of intestinal inflammation or hæmorrhage, the dose is from gr. ·075 to gr. ·15. The lithium salt is to be recommended in doses of grs. 1-3 for those of gouty diathesis. The iodate of mercuric oxide when dissolved in iodide of potassium constitutes a good hypodermic injection for syphillis. The results are apparently rapid, and attended with less stomatitis than is usual with the salts of mercury. The iodate of quinine in doses of ½ to 15 grains is an active substitute for the sulphate. It is stated that the iodate of codeine is a more efficient sedative and analgesic than the usual salts employed ; the hypodermic injection should contain about gr. ⅓. The iodate of hyoscine is one of the most powerful drugs known to medical science ; it appears to be far more rapid and effective in its action than either the hydrochlorate, the iodide, or the bromide, the dose being about $\frac{1}{400}$ grains for hypodermic use. The iodate of atropine has the advantage over other salts of this alkaloid in having a more rapid and evanescent effect, and, further, in possessing greater resistance to bacterial growth, so that no adventitious antiseptic need be added to the ordinary solution, which for ophthalmic use should be of about ½ per cent. strength.

* D. Med. Wenschrft., Sept., 1895.

